# Role of PKM2 Tetramer in Modulating Endothelial Mitochondrial Dysfunction in a Rat Model of Chronic Thromboembolic Pulmonary Hypertension

**DOI:** 10.33549/physiolres.935691

**Published:** 2026-02-01

**Authors:** Min-Xia YANG, Zhi-Zhu WU, Da-Wen WU, Mao-He CHEN, Qiu-Xia WU, Nan SHAO, Chao-Sheng DENG

**Affiliations:** 1 Division of Critical Care Medicine, First Affiliated Hospital of Fujian Medical University, Fuzhou, China; 2 Division of Critical Care Medicine, National Regional Medical Center, Binhai Campus of the First Affiliated Hospital, Fujian Medical University, Fuzhou, China; 3 Division of General Surgery, the Third People’s Hospital Affiliated to Fujian University of Traditional Chinese Medicine, Fuzhou, China; 4 Division of Respiratory and Critical Care Medicine, First Affiliated Hospital of Fujian Medical University, Fuzhou, China; 5 Division of Respiratory and Critical Care Medicine, National Regional Medical Center, Binhai Campus of the First Affiliated Hospital, Fujian Medical University, Fuzhou, China; 6 Division of Respiratory and Critical Care Medicine, Fujian Provincial Elderly Hospital, Fuzhou, China

**Keywords:** Chronic thromboembolic pulmonary hypertension, Lactic acid, Mitochondrial transcription factor A, Peroxisome proliferator-activated receptor γ coactivator 1α, Pyruvate kinase muscle

## Abstract

Dysfunction of pulmonary artery endothelial cells (PAECs) contributes to the pathogenesis of chronic thromboembolic pulmonary hypertension (CTEPH). However, the role of mitochondrial metabolism in this process remains unclear. The present study evaluated whether the tetrameric form of pyruvate kinase muscle isoform 2 (PKM2) regulates PAEC mitochondrial metabolism through peroxisome proliferator-activated receptor gamma coactivator 1 alpha (PGC-1α) and mitochondrial transcription factor A (mtTFA), thereby influencing arterial intimal remodeling in CTEPH. A CTEPH rat model was established by repeated injections of autologous thrombi. Activation of PKM2 tetramer expression was achieved through synthetic pyruvate kinase M2 activator (TEPP-46) administration. Pulmonary artery pressure (PAP), thrombus pathology, and protein expression levels of PKM2, mtTFA, and PGC-1α were assessed. Plasma lactate concentrations and tumor necrosis factor alpha (TNF-α) levels were measured. Rats with CTEPH demonstrated thrombotic obstruction, elevated PAP, and reduced expression of the PKM2 tetramer, mtTFA, and PGC-1α. Treatment with TEPP-46 was associated with a reduction in thrombus burden, lower PAP, and restoration of mitochondrial protein expression, accompanied by decreased lactate concentrations and TNF-α levels. In the CTEPH rat model, increased inflammation and elevated lactate concentrations were observed, along with decreased expression of mtTFA and PGC-1α in the pulmonary artery intima, which is indicative of mitochondrial dysfunction. The PKM2 tetramer may play a role in modulating PAEC mitochondrial function, reducing pulmonary artery pressure, and improving pulmonary arterial intimal remodeling in CTEPH.

## Introduction

Chronic thromboembolic pulmonary disease (CTEPD) is defined as persistent respiratory distress or limited physical activity lasting more than three months after the completion of standardized anticoagulant therapy for acute pulmonary embolism (APE), with or without pulmonary hypertension (PH) at rest. When CTEPD is accompanied by pulmonary arterial hypertension, the condition is classified as chronic thromboembolic pulmonary hypertension (CTEPH) [1,2]. CTEPH is a rare disorder associated with a high mortality rate [3].

Pyruvate plays a central role in the cellular metabolic network [4]. The function of pyruvate kinase muscle isoform 2 (PKM2) is determined by its structural configuration [5]. The tetrameric form of PKM2 is primarily located in the cytoplasm and possesses glycolytic activity that supports adenosine triphosphate (ATP) production [6]. In podocytes, the tetrameric configuration of PKM2 has been demonstrated to inhibit mitochondrial dysfunction induced by hyperglycemia, indicating that this configuration regulates energy metabolism through modulation of mitochondrial function [7]. Previous studies have demonstrated that rats with CTEPH exhibit elevated lactate concentrations, abnormal PKM2 expression in the pulmonary artery intima, and disrupted energy metabolism in endothelial cells [8]. These findings indicate an important role of PKM2 in the pathophysiology of CTEPH. However, the specific role of the PKM2 tetramer in endothelial mitochondrial dysfunction, as well as its association with endothelial metabolic reprogramming in CTEPH remains unclear. The present study established a CTEPH rat model and administered the PKM2 tetramer activator TEPP-46 to evaluate its effect on mitochondrial metabolic dysfunction in pulmonary artery endothelial cells in CTEPH.

## Methods

### Model establishment

The CTEPH rat model was established based on previously reported protocols [9]. Twenty-four healthy male Sprague-Dawley (SD) rats, aged 2 months and weighing 250 to 300 g, were obtained from the Experimental Animal Center of Fujian Medical University (Fuzhou, China). The animals were housed under controlled conditions with temperatures maintained at 20 °C to 24 °C and relative humidity at 65 % to 70 %. All experimental procedures were approved by the Animal Ethics Committee of Fujian Medical University (IACUC FJMU 2024-Y-0941) and conducted in accordance with the NIH Guide for the Care and Use of Laboratory Animals (Bethesda, MD, USA).

The rats were randomly assigned to three groups (n=8 per group) using the random number table method: control, CTEPH, and CTEPH + TEPP-46. The CTEPH group received autologous blood clot injections into the left external jugular vein, repeated on days 4 and 9 following the initial injection. Thrombi were prepared one day prior to injection by suspending 32±6 cylindrical blood clots (1 mm diameter, 2–3 mm length) in 1 ml saline, which was infused into the left external jugular vein at 0.2 ml/min. Control group rats underwent the same procedure but received an equivalent volume of saline instead of thrombi. The CTEPH + TEPP-46 group received the same thrombus protocol as the CTEPH group, with the addition of TEPP-46 (150 mg/kg/day) administered *via* gavage for 2 weeks.

All groups were administered penicillin (10,000 U/kg/day) for three days post-surgery to prevent bacterial infection. In addition, tranexamic acid (TXA, 200 mg/kg/day, intraperitoneally) was administered once daily to inhibit endogenous fibrinolysis. Animals were monitored for two weeks before specimen collection.

### Pulmonary artery pressure measurement

Following anesthesia, each rat was placed in the supine position on a small animal operating table. The skin of the right anterior cervical area was prepared and disinfected. A longitudinal incision was made from the right oral commissure toward the midclavicular line. Subcutaneous tissues were separated to isolate the right external jugular vein. A ligature was placed at the distal end of the vein, and a slipknot was left at the proximal end, secured with a vascular clamp. A V-shaped incision was made between the two ligatures. A polyethylene catheter connected to a PowerLab C multi-channel physiological recorder was prefilled with heparinized saline for baseline calibration. The catheter was inserted into the external jugular vein, and insertion progress was monitored *via* pressure waveform changes. Catheter positioning was confirmed by the characteristic right ventricular pressure waveform [9].

### Sample collection

Following pulmonary artery pressure measurement, 3 to 5 ml of blood was collected from the jugular vein. Plasma was separated by centrifugation, aliquoted into cryogenic vials, and stored at −80 °C. Rats were euthanized by exsanguination. For lung tissue collection, thoracotomy was performed, and the lungs were gently removed. Pulmonary arteries were flushed with physiological saline. Half of the lung tissue was fixed in 10 % formalin, embedded in paraffin, and processed for hematoxylin and eosin (H&E) staining and immunohistochemistry. The remaining lung tissue was used for pulmonary artery dissection, stored at −80 °C, and analyzed by Western blotting.

### Image analysis

Lung tissue samples were fixed in 10 % formaldehyde for 24 h, embedded in paraffin, and stained with H&E. Pulmonary arteries were examined using an optical microscope (Leica DMI3000M, Germany) and a digital medical image analysis system.

### Right ventricular hypertrophy index (RVHI)

The heart was excised, and the atria and residual blood vessels were removed. The free wall of the right ventricle (RV) was separated from the pulmonary artery outlet. The RV, left ventricle (LV), and interventricular septum (S) were dried on filter paper and weighed separately. The RVHI was calculated as: RVHI = RV/(LV + S).

### Plasma lactate and TNF-αconcentrations

Plasma lactate concentrations were determined using an L-Lactic Acid (LA) Colorimetric Assay Kit (Elabscience Biotechnology Co., Ltd., Houston, TX, USA) according to the manufacturer’s protocol. Plasma tumor necrosis factor alpha (TNF-α) concentrations were measured by enzyme-linked immunosorbent assay (ELISA) (SOLARBIO, China) following the manu-facturer’s instructions.

### Immunohistochemistry

Pulmonary artery sections underwent dewaxing and rehydration using a graded alcohol series. After blocking and antigen retrieval, sections were incubated with rabbit anti-rat PKM2 tetramer, mtTFA, and PGC-1α polyclonal antibodies (1:50, 1:100, and 1:200 dilutions, respectively; Abcam, Cambridge, UK). Detection was performed using the biotin-streptavidin-horseradish peroxidase (HRP) system (ZSGB-BIO, Shanghai, China) according to the manufacturer’s instructions. Negative controls were prepared by omitting the primary and secondary antibodies.

### Western blot analysis

Protein expression levels of PKM2 tetramer, mtTFA, and PGC-1α were evaluated *via* Western blotting. Protein concentrations were determined by the Lowry assay. Proteins were separated by sodium dodecyl sulfate-polyacrylamide gel electrophoresis (SDS-PAGE), transferred to membranes, and blocked with bovine serum albumin (BSA). Membranes were incubated overnight at 4 °C with anti-rat PKM2 tetramer, mtTFA, and PGC-1α antibodies (1:500, 1:500, and 1:1000 dilutions, respectively; Abcam). Subsequently, membranes were incubated with horseradish peroxidase (HRP)-conjugated rabbit secondary antibodies for 2 h. Signals were detected using enhanced chemilumi-nescence reagents, and protein bands were analyzed with LabWorks image analysis software (Gene Company Limited, Hong Kong, China).

### Statistical analysis

Statistical analyses were conducted using SPSS version 24.0 (IBM, Armonk, NY, USA). Data with a normal distribution are expressed as mean ± SD. Group comparisons were performed using analysis of variance, and Pearson’s correlation coefficients were used to assess relationships between variables. A p<0.05 was considered statistically significant.

## Results

### Pulmonary artery pressure and RVHI in different groups of rats

**Fig 1:**
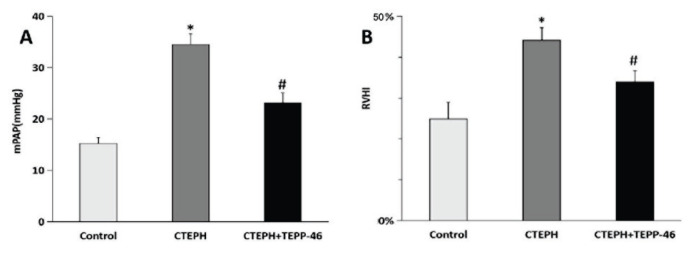
Mean Pulmonary artery pressure (mPAP) and Right ventricular hypertrophy index in rat groups. * Significant difference relative to the control group (p<0.05); **^#^** significant difference relative to the CTEPH group (p<0.05). CTEPH = chronic thromboembolic pulmonary hyper-tension.

A CTEPH rat model was successfully established through repeated injections of autologous thrombi. The mean pulmonary artery pressure (mPAP) in the CTEPH group (34.50±2.04 mmHg) was significantly higher than that in the control group (15.21±1.18 mmHg; p<0.05). Compared with the CTEPH group, the mPAP in the CTEPH + TEPP-46 group (23.09±1.94 mmHg) was significantly reduced (p<0.05; Fig. 1A). The right ventricular hypertrophy index (RVHI), calculated as RV/(LV + S), was significantly elevated in the CTEPH group (44.18±3.07 %) compared with the control group (24.90±4.06 %; p<0.05). Following TEPP-46 treatment, the RVHI decreased to 33.99±2.68 %, which was significantly lower than that in the CTEPH group (p<0.05; Fig. 1B).

### Pathological changes in the CTEPH rat model

**Fig. 2:**
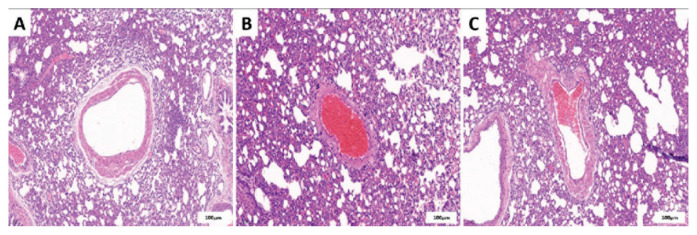
Histopathological changes in the pulmonary artery intima of rats in each group (hematoxylin-eosin staining). (**A**) Control group; (**B**) CTEPH group; (**C**) CTEPH + TEPP-46 group. CTEPH = chronic thrombo-embolic pulmonary hyper-tension.

Histological examination revealed that pulmonary vessels in the control group were free of thrombus obstruction (Fig. 2A). In the CTEPH group, unlysed thrombi with a dense structure and predominance of red blood cells was observed, along with nucleated cell infiltration (Fig. 2B). In the CTEPH + TEPP-46 group, thrombi appeared looser in structure, predominantly composed of red blood cells, with markedly fewer nucleated cells. The vascular lumen was not completely occluded (Fig. 2C).

### Plasma lactate and TNF-α concentrations in rats

**Fig. 3:**
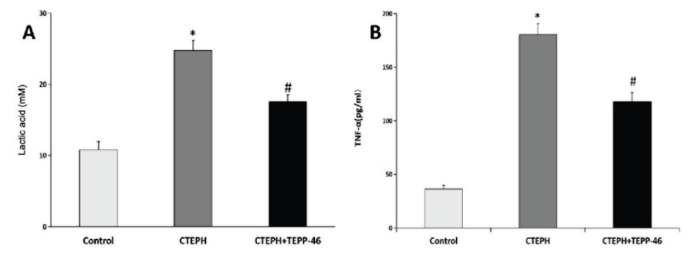
Plasma lactate and TNF-α concentrations in rat groups. * Significant difference relative to the control group (p<0.05); **^#^** significant difference relative to the CTEPH group (p<0.05). CTEPH = chronic thrombo-embolic pulm-onary hypertension.

Plasma lactate (Fig. 3A) and TNF-α (Fig. 3B) levels were lowest in the control group and significantly elevated in the CTEPH group (p<0.05). TEPP-46 treat-ment significantly reduced both lactate and TNF-α levels compared with the CTEPH group (p<0.05).

### Immunohistochemical expression of PKM2 tetramer, mtTFA, and PGC-1α

**Fig. 4:**
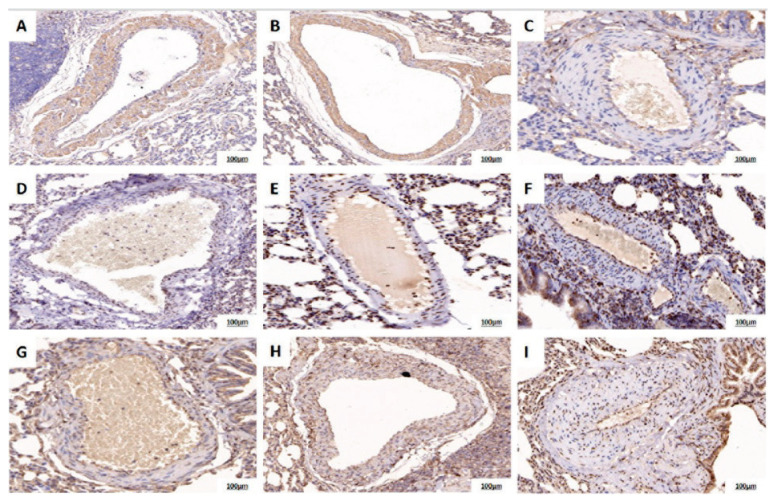
Immunohistochemical expression of PKM2 tetramer, mtTFA, and PGC-1α in pulmonary arteries of rat groups. (**A–C**) PKM2 tetramer; (**D–F**) mtTFA; (**G–I**) PGC-1α. A, D and G are control groups, B, E and H are CTEPH groups, and C, F and I are CTEPH+TEPP-46 groups. CTEPH = chronic thromboembolic pulmonary hypertension.

Immunohistochemistry revealed lower expression of PKM2 tetramer, mtTFA, and PGC-1α in the pulmonary artery intima of CTEPH rats compared with both the control and CTEPH + TEPP-46 groups (Fig. 4).

### Western blot expression of PKM2 tetramer, mtTFA, and PGC-1α proteins

**Fig. 5:**
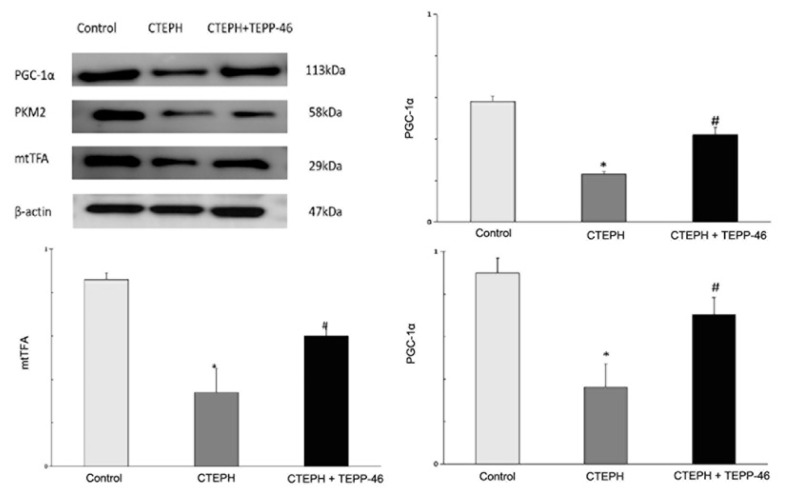
PKM2 tetramer, mtTFA, and PGC-1α protein expression in rat pulmonary artery intima. * Significant difference relative to the control group (p<0.05); **^#^** significant difference relative to the CTEPH group (p<0.05). CTEPH = chronic thromboembolic pulmonary hypertension.

Western blot analysis demonstrated reduced expression of PKM2 tetramer, mtTFA, and PGC-1α proteins in the CTEPH group relative to controls. TEPP-46 treatment increased the expression of these proteins compared with the untreated CTEPH group (Fig. 5).

### Correlation analysis

Pearson’s correlation analysis demonstrated a significant positive correlation between mPAP in the CTEPH group and the expression levels of PKM2 tetramer, mtTFA, and PGC-1α proteins. Detailed correlation values are presented in Table 1.

## Discussion

Following APE, thrombi may become insoluble and undergo organization, leading to chronic pathological changes and pulmonary vascular remodeling. This remodeling is characterized by vascular stenosis or occlusion, increased pulmonary vascular resistance (PVR), progressive elevation of pulmonary artery pressure, and ultimately right ventricular hypertrophy and failure – a condition defined as CTEPH. As a severe late complication of APE, CTEPH has drawn considerable clinical attention due to its poor therapeutic outcomes, high mortality, and disability rates [10–12]. Despite extensive research, its pathogenesis remains incompletely understood, particularly the regulatory mechanisms underlying pulmonary artery endothelial cells (PAECs) dysfunction after pulmonary thromboembolism. Clarifying these mechanisms remains a critical and contested issue in pulmonary vascular disease, including pulmonary embolism and pulmonary arterial hypertension.

Drawing on over a decade of experience in CTEPH animal modeling, this research group previously established a rat pulmonary embolism model that replicates the pathophysiology of CTEPH [9,13]. In the present study, thrombus dissolution efficiency in rats was found to be closely related to the size of the injected thrombi. Larger thrombi were less prone to dissolution, more likely to persist and obstruct blood vessels, and more readily underwent chronicization. Through methodological refinements – including increasing thrombus size and optimizing injection techniques – the constructed model demonstrated significantly elevated pulmonary artery pressure. Histopathological evaluation revealed unlysed thrombi obstructing pulmonary vessels with distal microvascular remodeling. Treatment with TEPP-46 resulted in a looser thrombus structure and decreased pulmonary artery pressure, indicating therapeutic potential.

Cellular metabolism is a critical determinant of endothelial cell phenotype and function [14]. PAEC dysfunction, driven by factors such as aberrant energy metabolism and inflammatory responses, may initiate pathological remodeling of the pulmonary artery intima during pulmonary arterial hypertension. Lactate, a byproduct of glucose metabolism, serves as a metabolic biomarker; elevated lactate levels indicate disordered energy metabolism, whereas reductions indicate improved tissue oxygenation and metabolic function [15]. TNF-α, a potent pro-inflammatory cytokine, promotes cell necrosis and vascular remodeling. Elevated TNF-α has been documented in both patients with CTEPH and animal models, where it correlates with disease severity, altered pulmonary hemodynamics, and intimal changes [16].

In this study, lactate and TNF-α concentrations were significantly higher in the CTEPH group compared with controls, indicating metabolic disruption and inflammatory infiltration. TEPP-46 treatment reduced both parameters, supporting a role for PKM2 tetramers in modulating energy metabolism and inflammatory pathways in CTEPH.

Pyruvate kinase (PK) is a key glycolytic enzyme that catalyzes the conversion of phosphoenolpyruvate to pyruvate, generating ATP. It regulates the cellular metabolic phenotype and exists in most cell types as either the M1 or M2 isoform, or both [4]. Recent therapeutic strategies have focused on targeting and activating the tetrameric form of PKM2 to restore normal metabolic activity. Activation of PKM2 tetramers by specific small-molecule activators reduces the accumulation of glycolytic intermediates that support tumor cell proliferation [17]. TEPP-46, a synthetic PKM2 activator, binds to an interface pocket on the PKM2 subunit, stabilizing the tetrameric conformation. This tetrameric PKM2 has enzymatic activity comparable to PKM1 and significantly attenuates tumorigenicity in murine cancer models [18,19].

Beyond oncology, PKM2 tetramers have been implicated in mitochondrial protection. Studies have demonstrated that tetrameric PKM2 can inhibit hyperglycemia-induced mitochondrial dysfunction in podocytes, thereby mitigating diabetic glomerulopathy progression [7]. Through modulation of mitochondrial function, PKM2 tetramers influence overall cellular energy metabolism.

Mitochondrial biosynthesis is governed by key transcriptional regulators, including peroxisome proliferator-activated receptor gamma coactivator 1-alpha (PGC-1α), nuclear respiratory factors 1 and 2 (NRF1, NRF2), and mitochondrial transcription factor A (mtTFA) [20,21]. PGC-1α orchestrates transcriptional programs that enhance mitochondrial quality, enabling tissues to adapt to elevated energy demands [20,22]. MtTFA is critical for regulating mitochondrial DNA copy number and directly influences mitochondrial biogenesis. Silencing mtTFA expression using lentiviral vectors or small interfering RNA markedly inhibits mitochondrial biogenesis [23].

TEPP-46 has been reported to promote PKM2 tetramer formation, upregulate mtTFA and PGC-1α expression by inhibiting activation of the PI3K/Akt signaling pathway, and enhance mitochondrial biosynthesis in macrophages. This process reduces the release of inflammatory mediators and promotes endotoxin tolerance [20,24–26]. In the present study, PKM2 tetramer, mtTFA, and PGC-1α expression levels were significantly decreased in the CTEPH group and were negatively correlated with pulmonary artery pressure. Moreover, PKM2 tetramer expression in the pulmonary artery intima was positively correlated with mtTFA and PGC-1α levels. TEPP-46 treatment reduced thrombus burden in the pulmonary vascular bed, lowered pulmonary artery pressure, and increased the expression of PKM2 tetramer, mtTFA, and PGC-1α. These findings suggest that CTEPH may share pathogenic mechanisms with tumor biology, specifically mitochondrial metabolic dysfunction. Collectively, the results support a key role for PKM2 tetramers in regulating abnormal energy metabolism in PAECs in CTEPH, potentially through the modulation of mitochondrial function.

This study has several limitations. First, it evaluated the effects of PKM2 tetramer on pulmonary arterial energy metabolism in CTEPH rats only at the tissue level and did not directly investigate its role and underlying mechanisms in PAECs. Although pulmonary arterial hypertension was observed in the CTEPH rat model, the absence of functional assessments of cellular metabolism limits extrapolation of these findings to human disease, warranting further investigation. Second, TEPP-46 and thrombus were administered simultaneously, making the intervention more akin to primary prevention rather than treatment of established CTEPH. Future studies should refine the experimental design to differentiate between therapeutic and preventive effects by varying the timing of drug administration. Moreover, subsequent research should focus on delineating the specific signaling pathways linking PKM2 tetramer to mitochondrial biogenesis markers, such as mtTFA and PGC-1α, in PAECs during different stages of CTEPH progression.

## Conclusions

In CTEPH rats, elevated lactate and TNF-α levels are accompanied by reduced expression of mtTFA and PGC-1α, key markers of mitochondrial biogenesis, as well as decreased PKM2 tetramer levels in the pulmonary artery intima. These alterations contribute to mitochondrial metabolic dysfunction. Enhancing PKM2 tetramer expression may upregulate mtTFA and PGC-1α, thereby ameliorating mitochondrial metabolic dysfunction in PAECs, improving endothelial function, and ultimately attenuating pulmonary artery intimal remodeling. Thus, targeting PKM2 tetramer expression represents a promising therapeutic strategy for preventing or slowing the chronic progression of pulmonary embolism to CTEPH.

## Figures and Tables

**Table 1:** Correlation between mean pulmonary artery pressure (PAP) and PKM2 tetramer, mtTFA, and PGC-1α protein levels in the CTEPH group.

	PAP and PKM2 tetramer	PAP and mtTFA	PAP and PGC-1α
*r*	−0.927	−0.930	−0.940
*p*	0.001	0.001	0.001

## Abbreviations

APE: Acute pulmonary thromboembolism
CTEPD: Chronic thromboembolic pulmonary disease
CTEPH: Chronic thromboembolic pulmonary hypertension
LV: Left ventricle
mtTFA: Mitochondrial transcription factor A
NRF1: Nuclear respiratory factor 1
PAEC: Pulmonary artery endothelial cell
PAH: Pulmonary arterial hypertension
PE: Pulmonary embolism
PGC-1α: Peroxisome proliferator-activated receptor γ coactivator 1α
PKM: Pyruvate kinase muscle
PTE: Pulmonary thromboembolism
PVR: Pulmonary vascular resistance
RV: Right ventricle
RVHI: Right ventricular hypertrophy index
TNF-α: Tumor necrosis factor-α
